# Multiplex Detection of *Salmonella* spp., *E. coli* O157 and *L. monocytogenes* by qPCR Melt Curve Analysis in Spiked Infant Formula

**DOI:** 10.3390/microorganisms8091359

**Published:** 2020-09-04

**Authors:** Sarah Azinheiro, Joana Carvalho, Marta Prado, Alejandro Garrido-Maestu

**Affiliations:** 1 Food Quality and Safety Research Group, International Iberian Nanotechnology Laboratory, Av. Mestre José Veiga s/n, 4715-330 Braga, Portugal; sarah.azinheiro@inl.int (S.A.); joana.carvalho@inl.int (J.C.); marta.prado@inl.int (M.P.); 2 College of Pharmacy/School of Veterinary Sciences, University of Santiago de Compostela, Campus Vida, E-15782 Santiago de Compostela, Spain

**Keywords:** multiplex qPCR, melting analysis, food analysis, *Listeria monocytogenes*, *Salmonella* spp., *E coli* O157, non-competitive internal amplification control (NC-IAC)

## Abstract

Food poisoning continue to be a threat in the food industry showing a need to improve the detection of the pathogen responsible for the hospitalization cases and death. DNA-based techniques represent a real advantage and allow the detection of several targets at the same time, reducing cost and time of analysis. The development of new methodology using SYBR Green qPCR for the detection of *L. monocytogenes*, *Salmonella* spp. and *E. coli* O157 simultaneously was developed and a non-competitive internal amplification control (NC-IAC) was implemented to detect reaction inhibition. The formulation and supplementation of the enrichment medium was also optimized to allow the growth of all pathogens. The limit of detection (LoD) 95% obtained was <1 CFU/25 g for *E. coli* O157, and 2 CFU/25 g for *Salmonella* spp. and *L. monocytogenes* and regarding the multiplex detection a LoD 95% of 1.7 CFU/25 g was observed. The specificity, relative sensitivity and accuracy of full methodology were 100% and the use of the NC-IAC allowed the reliability of the results without interfering with the sensitivity of the methodology. The described study proved to obtain results comparable to those of probe-based qPCR, and more economically than classical high resolution melting qPCR, being both important aspects for its implementation in the food industry.

## 1. Introduction

The hazard associated with food contamination continues to be a worldwide human health serious concern [1,2]. The number of outbreaks related with food, and water contamination, has not shown any significant decrease in the past four years, and according with the annual report of the European Food Safety Authority (EFSA) and the European Center for Disease Control and Prevention (ECDC), in 2016 an increase in the general trend was observed compared with 2015. Among the causative agents of foodborne illness, pathogenic bacteria appear as the most problematic. *Salmonella* spp. continues to be one of the most prevalent in food products with 94,530 confirmed human cases in 2016, only behind *Campylobacter* spp. An increase in confirmed cases has also been observed in other foodborne illnesses, such as for listeriosis and those related with Shiga toxin-producing *Escherichia coli* (STEC) infections. Listeriosis, caused by *L. monocytogenes*, is one of the most severe zoonosis as has the highest mortality rate, reaching 16.2% in 2016, and exhibited a 9.3% increase in the number of cases reported. Regarding the STEC, an increase of 8.3% in confirm cases compared to 2015 was observed, being O157 the most commonly reported serotype [3].

Despite the efforts to control the food production chain, difficulties in tracking outbreaks, and detection of the contamination source before human infection remain [4,5]. Traditional methodologies take more than one week to have the final result, and independent protocols must be applied for each pathogen. This approach is not compatible with the intense production existing nowadays. Fast and reliable analytical methods are needed by the industry, and control laboratories, to avoid potential illness and deaths. The development of molecular techniques based in DNA analysis, such PCR and particularly real-time PCR (qPCR), allow to reduce the time of analysis, decreasing hand-on steps allowing to handle several samples at the same time and detect different pathogens in a single analysis, being an advantage to reduce cost of reagents and labor needed [6].

The multiplexing in qPCR has been already reported in different studies [7,8,9,10]. The use of hydrolysis probes to monitor independently the amplification of each target increases the specificity, however the chemistry is more costly and requires the synthesis of specific oligonucleotides [11]. Melting analysis, using intercalating dyes, shows potential, for being a cost efficient high throughput methodology. The melting analysis is based in the behavior of double stranded DNA when submitted to increase of temperature. The temperature increase will dissociate the dsDNA molecule, depending in the length and the guanine-cytosine (GC) content of the DNA fragment. The value of the melting temperature, representing the temperature at which 50% of the DNA is single stranded, will identify the different fragments present in the reaction [12]. In qPCR, different intercalating dyes, such as SYBR Green, have been extensively used to monitor the amplification curve, but also to allow the visualization of melting curves of the different amplicons generated. In this way, the identification, and differentiation, between different targets in a same sample is possible. This approach was already tested for the detection of different foodborne pathogens, but also to differentiate between species or serovar closely related by phylogenetic and genetic criteria [13,14,15].

Therefore, the aim of the present study was the development and evaluation of a SYBR Green multiplex qPCR methodology for the simultaneous identification of *Salmonella* spp., *L. monocytogenes* and *E.coli* O157 in food samples, by melting curve analysis. Additionally, a non-competitive internal amplification control (NC-IAC) was co-amplified in the qPCR reactions to avoid false negative results due to reaction inhibition. Finally, the sample enrichment was also optimized to assure appropriate recovery of the pathogens of interest, and to have a faster and highly sensitive analysis. The novel method does not need expensive probes, or sophisticated intercalating dyes to perform melt curve analysis with a dedicated software, what simplifies the methodology and reduces the cost.

## 2. Materials and Methods

### 2.1. Bacterial Stains

*L. monocytogenes* (WDCM 00021), *E. coli* O157:H7 (WDCM 00014) and *S*. Typhimurium (WDCM 00031) were used as reference organisms for the evaluation of the methodology. Fresh cultures of each bacteria were prepared by resuspending a single colony in 4 mL of Nutrient Broth (NB, Biokar Diagnostics S.A., Allonne, France). The culture was incubated overnight (ON) at 37 °C, and to determine the concentration of bacteria used in each assay, ten-fold serial dilutions of the initial culture were made, in NB and plated on Tryptic Soy Agar (TSA) (Biokar Diagnostics S.A., Allonne, France) for *E.coli* O157:H7 and *S*. Typhimurium, and on Tryptic Soy Yeast Extract Agar (TSYEA, Biokar Diagnostics S.A., Allonne, France) for *L. monocytogenes*. The plates were incubate ON, at 37 °C.

### 2.2. Enrichment Optimization

The competition of growth between the different pathogens to be identified may influence the sensitivity of the methodology, for this reason, the enrichment step was optimized to allow the best performance. *L. monocytogenes* grows slower than the other two pathogens, thus the major concern was to increase its growth rate, and adjust the better conditions for higher competitiveness. The media formulation and the addition of several supplements, and temperature optimization were tested.

#### 2.2.1. Media Formulation

A growth study was perform to evaluate different modifications of modified TA10 broth (mTA10) describe by Garrido et al. [10] in *L. monocytogenes*., at two different temperature 30 °C and 35 °C. Three medias were tested, mTA10 (10 g/L Tryptose (Pronadisa, Torrejón de Ardoz, Spain), 5 g/L Beef extract (Scharlau Chemie S.A., Sentmenat, Barcelona, Spain), 5 g/L Yeast extract (Difco, BD & Co., East Rutherford, NJ, USA), 5 g/L NaCl, 3.4 g/L KH_2_PO_4_ and 19.3 g/L Na_2_HPO_4_), mTA10 replacing the KH_2_PO_4_ and Na_2_HPO_4_ by 8.5 g/L of 3-(N-morpholino)propanesulfonic acid (MOPS) and 13.7 g/L MOPS sodium salt, and also testing the addition of 0.5 g/L of glucose in mTA10-MOPS. All the chemicals, and the glucose, were acquired from Sigma-Aldrich (Sigma–Aldrich, St. Louis, MO, USA). To perform this growth study, 96 well-plates were used, 200 µL of each medium were inoculate with 2 µL of fresh bacterial culture prepared as described above, with a final concentration of 10^2^–10^3^ CFU. The absorbance at 600 nm was measured every 30 min during 24 h in a Microplate Reader (Synergy, BioteK H 1, Winooski, VT, USA), with constant agitation.

#### 2.2.2. Media Supplementation

After choosing the medium and temperature which allowed better growth of *L. monocytogenes* separately, the use of several supplements, such as 50 mL/L Laked Horse Blood (Oxoid, United Kingdom), 4 mL/L *Campylobacter* Growth Supplement (Oxoid, UK), 225 µL/225 mL Half Fraser Supplement (Biokar Diagnostics S.A., Allonne, France) and as well the combinations of these compounds together were tested in mixed cultures with the three bacteria simultaneously. The mixed cultures were prepared in 50 mL of mTA10-MOPS supplemented with the different compounds, and were inoculate with 10–10^2^CFU of each target species and incubated at 35 °C, 24 h. After the incubation, ten-fold serial dilutions of the culture were plated in different selective media, such as COMPASS (Biokar diagnostics S.A., Allonne, France), CHROMagar™ *Salmonella* Plus (CHROMagar, Paris, France) and Tryptone Bile X-Glucuronide Agar (TBX) for the quantification of *L. monocytogenes*, *Salmonella* spp. and *E.coli* O157, respectively. Plates of CHROMagar™ *Salmonella* Plus and COMPASS were incubate at 37 °C and TBX plate at 44 °C, ON.

### 2.3. Growth Kinetic Model and Statistical analysis

Microbial growth kinetics were modelled using a logistic equation, reparametrized according to Zwietering et al. [16] to explicitly show those parameters with a biological meaning:

OD(t) = OD”max”/(1 + e^(((4 μ”max”)/OD”max” (λ − t) + 2)))(1)

where OD(t) represents the optical density, measured at 600 nm, at time t, ODmax represents the maximum optical density, µmax is the maximum specific growth rate in h-1, and λ is the lag time in h. Data fitting, assessment of the model parameters significance (Student *t*-test; α = 0.05), and consistency of the mathematical model (Fisher’s F test; *p* < 0.05) were performed with Mathematica 9 (Wolfram Research, Inc., Witney, UK).

After values adjusted with the model, the results were submitted to Mann–Whitney U test. Statistical analysis was performed with GraphPad Prism 5.0 software in order to know if significant differences were observed (*p* < 0.05) in the maximum absorbance, maximum rate of growth or lag phase of the *L. monocytogenes* growth with the different medium tested.

### 2.4. Food Spiking

Infant milk formula was selected as reference food matrix for the evaluation of the developed methodology. Twenty-five mL of milk sample were inoculated at different contamination levels of each target microorganism, and diluted 1:10 with 225 mL of mTA10-MOPS. The matrix was homogenized in a Stomacher 400 Circulator, at 230 rpm, for 30 s. The pre-enrichment was performed at 35 °C for 24 h and viable counts, prior to inoculation, of each pathogen were obtained as described in M&M 2.1.

### 2.5. DNA Extraction

DNA extraction was performed following two different protocols. To obtain DNA from pure cultures the bacteria were thermally lysed, however for the DNA extraction from food samples, enzymatic lysis was perform.

#### 2.5.1. Pure Culture

The evaluation of the inclusivity and exclusivity of the qPCR was performed with pure DNA extracts of the species detailed in Table 1. Pure cultures of the strains were obtained as described in M&M 2.1, and the DNA extraction was performed by thermal lysis. Resuming, the cultures were first centrifuged at 16,000× *g* for 5 min to concentrate the bacteria, the supernatant was removed, the pellet resuspended in 1 mL of TE 1X (10 mM Tris-HCl, 1mM EDTA (Sigma–Aldrich, St. Louis, MO, USA), pH 7.5) and centrifuged again in the same conditions. Again, the supernatant was discarded, the pellet was resuspended in 300 µL of TE 1X. This new bacterial suspension was incubated for 15 min at 99 °C, with constant agitation (1400 rpm), to lyse the cells in Thermomixer comfort (Eppendorf AG, Hamburg, Germany). Finally, the thermally lysed bacteria were centrifuged at 16,000× *g* for 5 min at 4 °C, to separate the DNA (supernatant) from the other cell debris (pellet). The supernatant was transferred to a clean tube, and stored at −20 °C until needed.

#### 2.5.2. Food Samples

The DNA extraction from spiked food samples was performed based on the Lysis-GuSCN method described by Kawasaki et al., [17] with some modifications. Briefly, 1 mL of the pre-enriched sample was centrifuged at 380× *g*, for 2 min to pellet any large food particles, and the supernatant was transferred to a new tube, which was centrifuged at 16,000× *g* for 5 min. This time the supernatant was discarded, and the pellet resuspended in PBS. The suspension was centrifuged again under the same conditions. The supernatant was discarded again, and the resulting pellet was treated with 200 µL of an enzymatic solution containing 1 mg/mL of achromopeptidase (Sigma–Aldrich, St. Louis, USA) and 20 mg/mL of lysozyme (Sigma–Aldrich, St. Louis, USA) in TE 2X with 1.2% of Triton X-100 (Sigma–Aldrich, St. Louis, MO, USA). The samples were incubated for 30 min at 37 °C, with constant agitation in a Thermomixer comfort (1400 rpm). After the incubation, 300 µL of a solution containing 4 M of Guanidine isothiocyanate (Sigma–Aldrich, St. Louis, MO, USA) and 1% of Tween 20 (Sigma–Aldrich, St. Louis, USA) were added, and 400 µL of this solution were transfer to 400 µL of 100% isopropanol (Sigma–Aldrich, St. Louis, MO, USA), and centrifuged for 10 min at 16,000× *g*. The pellet was rinsed with 1 mL of 75% isopropanol, resuspended in 160 µL of sterile Milli-Q water, and incubated at 70 °C, for 3 min. Finally, before use, the DNA was separated from any remaining debris by a 5 min centrifugation at 16,000× *g* and 4 °C.

### 2.6. qPCR Reaction

Three sets of primers were designed and optimized to allow the detection of the three targets. The genetic targets chosen were *actA*, *fimA* and *rfbE* for *L. monocytogenes*, *Salmonella* spp. and *E. coli* O157, respectively. The primers were designed to have different melting temperatures in order to discriminate between the pathogens. To do so, the melting curve of the fragments obtained by the sets of primers designed were predicted and analyzed using uMELT online software (https://www.dna.utah.edu/umelt/umelt.html), to ensure the distinction between the melting peak generate. The primer design was performed using the free online software Primer3 [18]. NC-IAC was also introduced in the reaction to ensure a reliable result and avoid false negative results due to reaction inhibition. The DNA sequence of the NC-IAC was designed using to generate a random DNA fragment and this sequence was subsequently used as template for primer design as previously described [19]. The specificity of all primers was verified in silico with BLAST^®^ (Basic Local Alignment Search Tool, https://blast.ncbi.nlm.nih.gov/Blast.cgi?CMD=Web&PAGE_TYPE=BlastHome).

After optimization, the qPCR reaction was performed in final volume of 25 µL with 3 µL of template. The reaction was carried with the primer concentrations specified in Table 2, 1 µL of NC-IAC DNA (926 copies/µL), and 15 uL of PowerUp™ SYBR^®^ Green Master Mix (ThermoFisher, USA). The thermal profile consisted on a first step of UDG treatment at 50 °C for 2 min, followed by a hot-start activation of the polymerase at 95 °C during for 2 min, and 40 cycles of 95 °C for 15 s and 63 °C for 1 min. The melting curve stage was performed by heating at 95 °C for 15 s, cooling to 70 °C for 1 min, and increasing back to 95 °C with continuous increments of 0.015 °C/s. Once reached the final temperature it was kept for 15 s.

### 2.7. Evaluation

To ensure the reliability of the developed methodology, a complete evaluation was performed in two steps. First the evaluation of the qPCR reaction, and second the analysis of the full methodology with artificially contaminated samples.

#### 2.7.1. Evaluation of qPCR Assay (Efficiency, Dynamic Range, Inclusivity/Exclusivity)

Forty five different bacterial species/strains, detailed in Table 2, were used to test the inclusivity/exclusivity of qPCR reaction. The dynamic range was determine also for the design primers and qPCR reaction, for each bacterium separately, using ten-fold serial dilutions of DNA extracted from a pure culture of each reference strain. Additionally the amplification efficiency (simplex and multiplex) of the reaction was determined using ten-fold serial dilutions of pure, or a mixture, of the DNA extracts. The efficiency was automatically calculated by the thermocycler software, using the following formula: *e* = [10^(−1/slope)^] – 1, where “*e*” is the efficiency [22].

#### 2.7.2. Evaluation of the Full Methodology

To test the capability of the methodology to detect the target microorganisms in real food samples, the limit of detection (LoD) of the methodology was evaluated for the presence of the three bacteria in different contamination levels. The LoD was determined analyzing the probability of detection (POD) using PODLOD calculation program, version 9 [23] to predict the probability of detection for each target. LoD_50_ and LoD_95_ were analyzed, which specify the smallest amount of each pathogen that can be detected with a probability of 50% and 95%, respectively. The evaluation of the LoD for each target pathogen was made spiking six samples for each contamination level until not being possible the detection, generating a false negative result.

Additionally, samples were classified as positive and negative agreement (PA/NA), and positive and negative deviation (PD/ND) comparing the result obtained after analysis with the expected results. Using these data the relative sensitivity, specificity, accuracy and (SE/SP/AC), positive and negative predictive values (PPV/NPV) and the Cohen’s kappa, or k, were calculated as previously described [10].

## 3. Results

### 3.1. Evaluation of mTA10 Medium Modifications and Supplements

The medium optimization for the multiplex detection by this methodology was made by two different approaches to evaluate modifications of mTA10 medium.

#### 3.1.1. Optimization of Buffering Agent and Temperature

**Figure 1:**
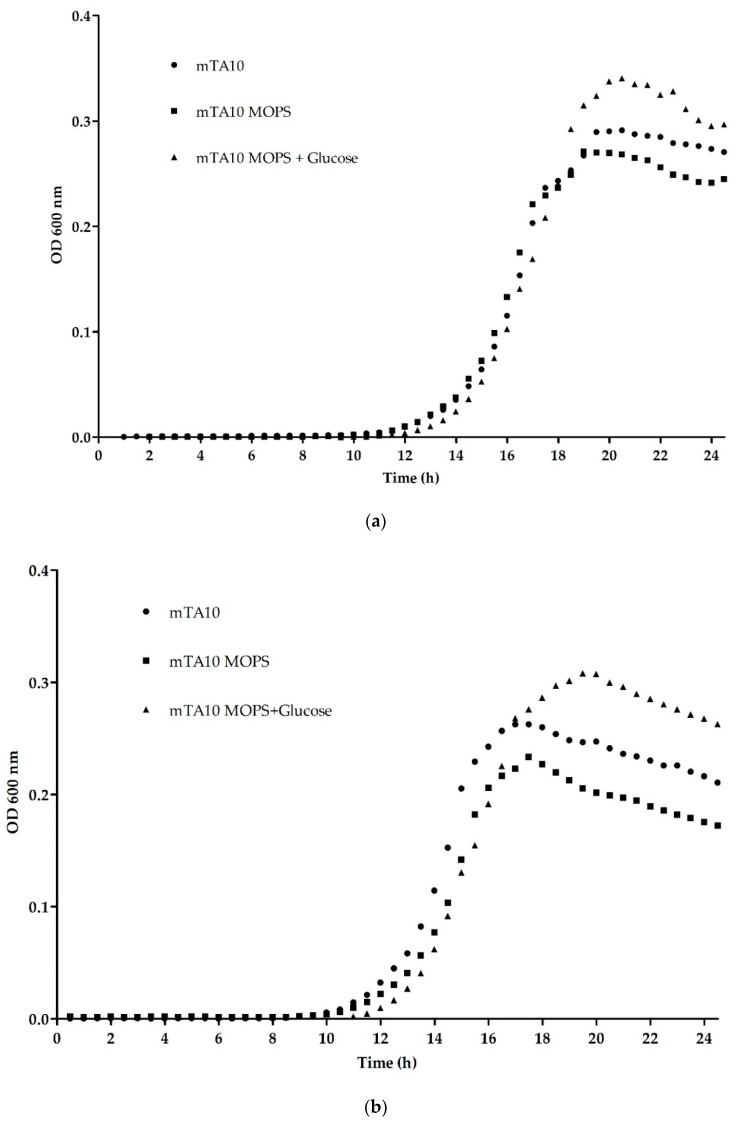

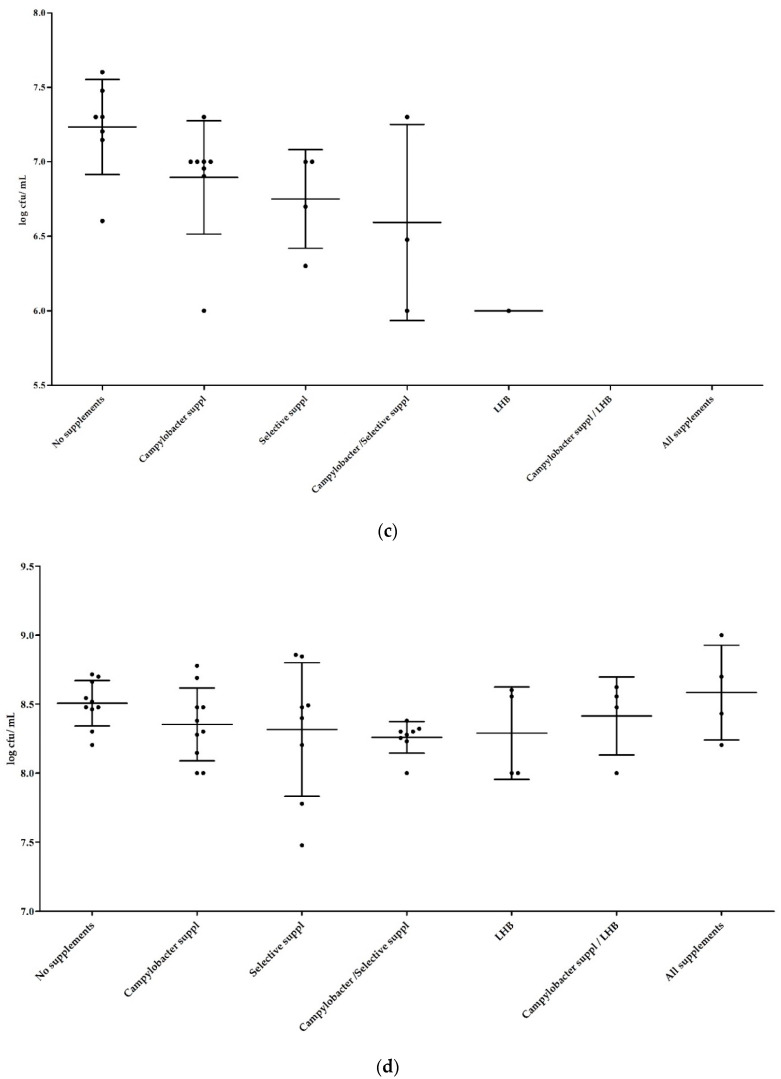

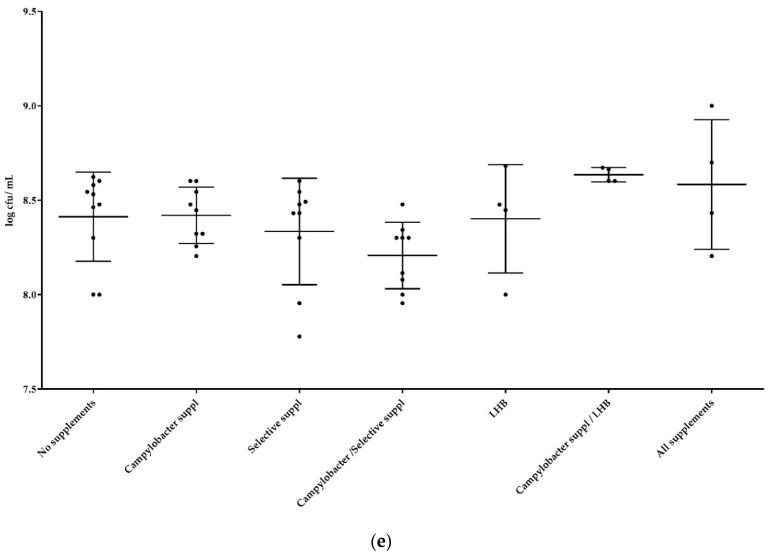
mTA10 medium optimization; (**a**,**b**) represent the growth kinetics at 30 °C and 35 °C respectively, of *L. monocytogenes* in mTA10, mTA10-MOPS and mTA10-MOPS with 0.5 g/L of glucose. (**c**–**e**) present the plating results of mix cultures of *L. monocytogenes*, *E. coli* O157 and *S*. Typhimurium after 24 h of incubation in mTA10 with the different supplements. *Campylobacter* suppl., indicates the addition of *Campylobacter* growth supplement; LHB, Laked Horse Blood; Selective suppl. refers to Half Fraser Supplement.

The substitution of the KH_2_PO_4_ and Na_2_HPO_4_ by MOPS to buffer mTA10 medium was evaluated by following the growth of *L. monocytogenes* for 24 h. Figure 1a,b represents the kinetic curves obtained and Table 3 shows the parameters given by the model. No statistically significant differences (*p* > 0.05) in lag phase, maximum OD_600_, growth rate between the use of the two different buffers were observed, attending to the result obtained by the Mann–Whitney U test. Two different temperatures were also analyzed to determine the best growth of the bacteria, and the results showed that 35 °C reduced the lag phase in approximately 1 h, being around 13 h instead of 14 h and allowed to reach the maximum OD_600_ more than 2 h before, compared with the results shown at 30 °C, but this difference are not statistically significant.

#### 3.1.2. Influence of Supplements in Mixed Cultures

Laked Horse Blood, *Campylobacter* Growth Supplement and Half Fraser Selective Supplement were chosen to evaluate their effect alone and combined in the growth of *L. monocytogenes*, *Salmonella* spp. and *E. coli* O157. The result presented in Figure 1c–e shows that Laked Horse blood and *Campylobacter* Supplement combined improve the growth of *Salmonella* spp., as while not statistically significant, the plate counts were higher with a relatively small SD. No major effects, promoting or inhibiting, were observed on the plate counts of *E. coli* O157. However, none of them were able to enhance the growth of *L. monocytogenes*, and actually had some detrimental effect even though it was not statistically significant. Figure 1c shows lower concentration of this bacterium after 24 h of incubation in mTA10-MOPS supplemented with all different compounds in co-culture. This effect is even higher with the addition of LHB, and its combination with any of the others, being the plate counts for these lower than 6 log CFU/mL.

### 3.2. Melt Peak Analysis

**Figure 2:**
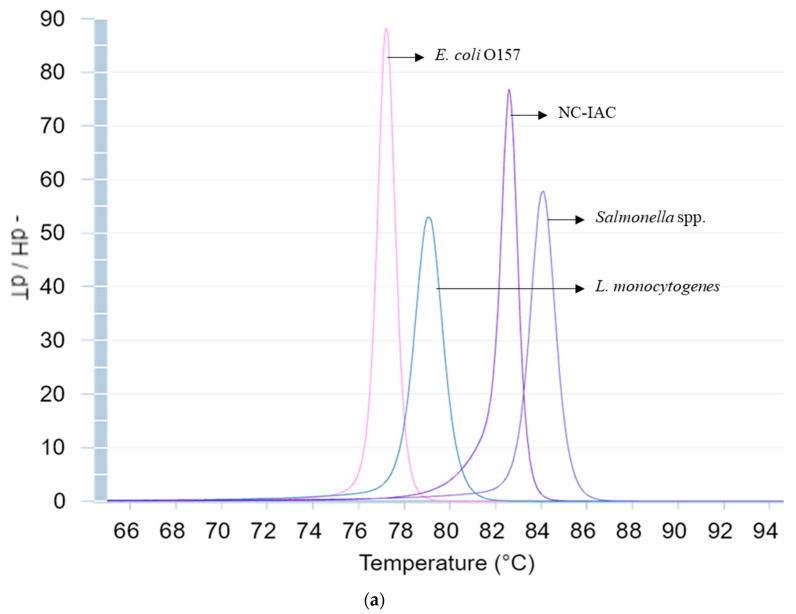

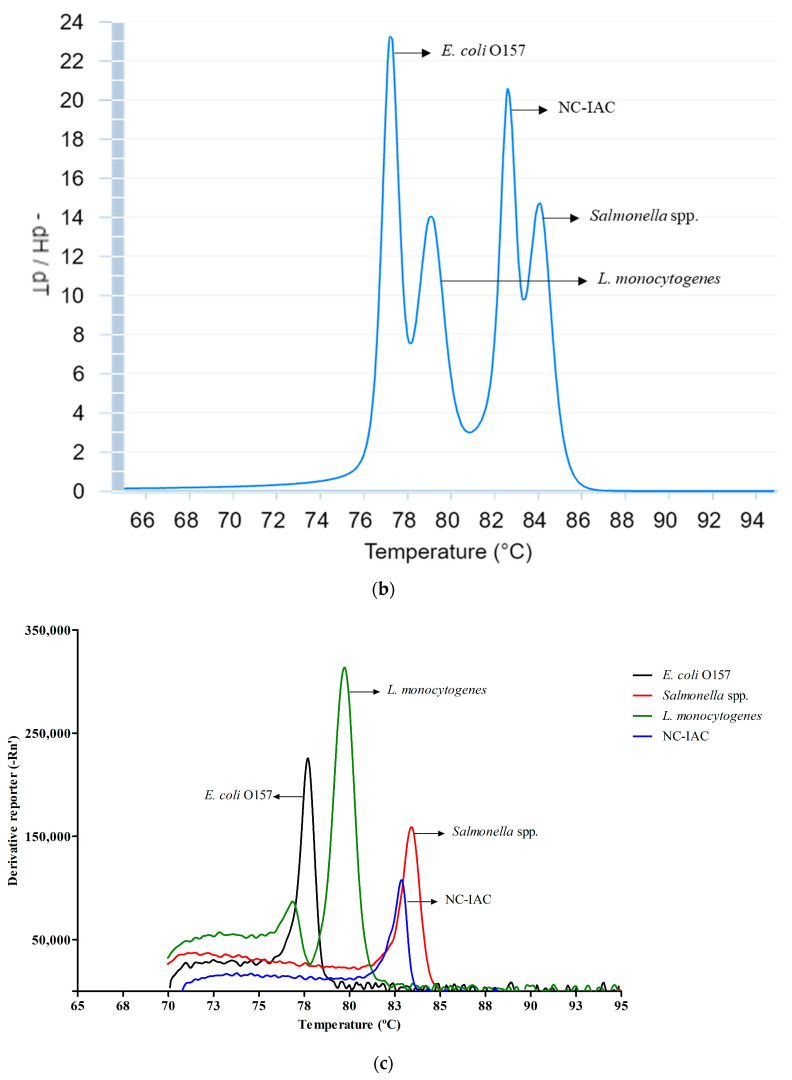

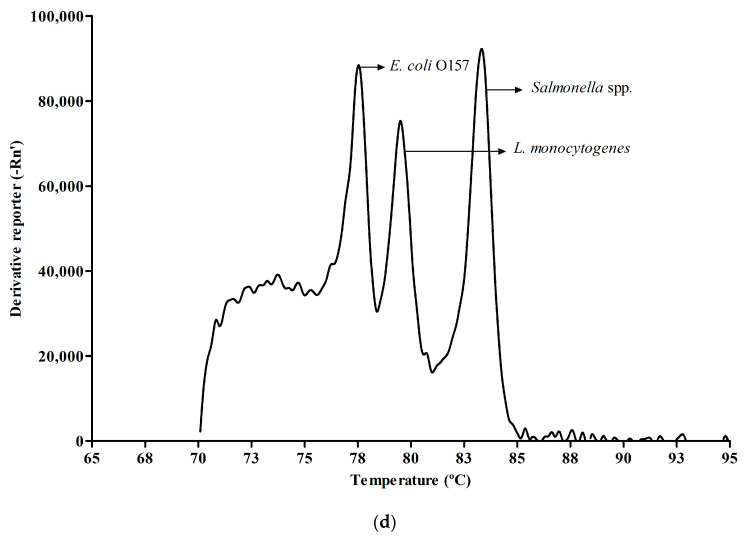
Predicted melting curves generated by uMELT online software in simplex (**a**) and multiplex (**b**) reaction and experimental melting curves obtained by pure culture in simplex (**c**) and by sample analysis in multiplex (**d**).

The theoretical values given by uMELT online software used for primers-design present a similar pattern when comparing with the peak obtained during the analysis of the samples. The predicted values are around 77.5, 79.5, 84 and 82.5 and the sample obtained an average melting peak of 77.45 ± 0.13, 79.47 ± 0.11, 83.20 ± 0.13, 82.67 ± 0.16 for the identification of *E. coli* O157, *L. monocytogenes*, *Salmonella* spp. and NC-IAC, respectively. Only *Salmonella* peak presented a lower temperature compared with the predicted, being closer to the peak which identifies the NC-IAC. Despite this rapprochement, it can clearly distinguish both the targets. The predicted and the experimental melting temperatures obtained with pure culture and within the sample analysis melting peaks are represented in Figure 2.

### 3.3. Evaluation of qPCR Reaction

To evaluate the performance of the optimized qPCR reaction with *actA*, *fimA*, *rfbE* and NC-IAC primers the inclusivity/exclusivity and efficiency were determined.

#### 3.3.1. Inclusivity/Exclusivity

Pure cultures of a total of 45 different strain were tested to evaluate the inclusivity/exclusivity of the qPCR and results are presented in Table 2. The inclusivity of the multiplex qPCR was evaluated with, 13 *Salmonella* spp., 18 *L. monocytogenes* and 1 *E. coli* O157, and all strains presented their corresponding meting peak. Regarding the exclusivity, 13 other bacteria were evaluated, including two other *Listeria* species and two *E. coli* strains. As expected, all non-target microorganism amplified with Cq values of 35.36 ± 0.81 with a melting peak of 82.78 ± 0.09, specific for NC-IAC amplification.

#### 3.3.2. Efficiency and Dynamic Range of qPCR in Simplex and Multiplex

**Figure 3:**
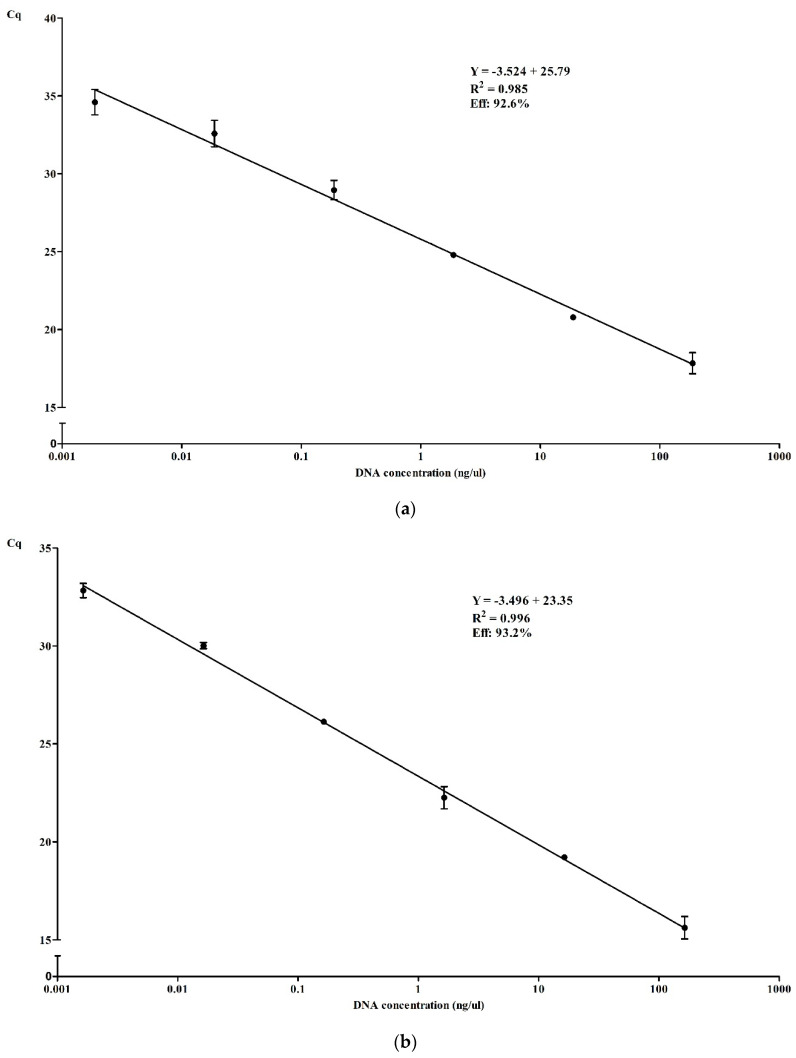

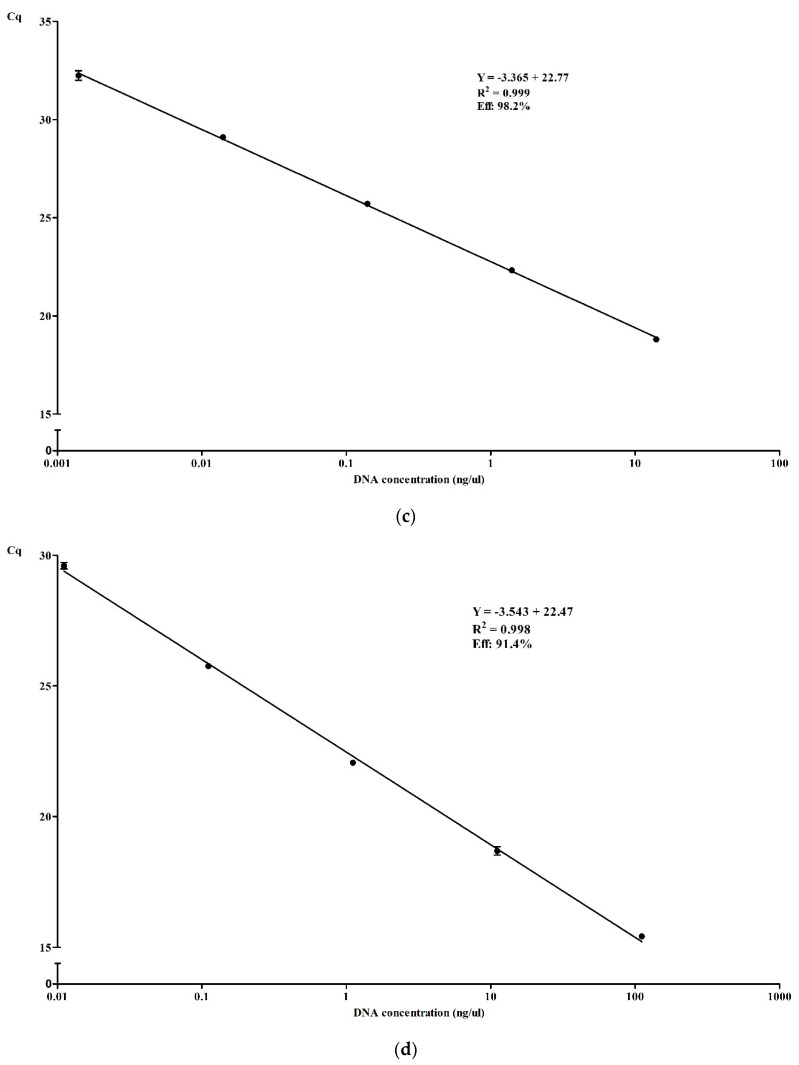

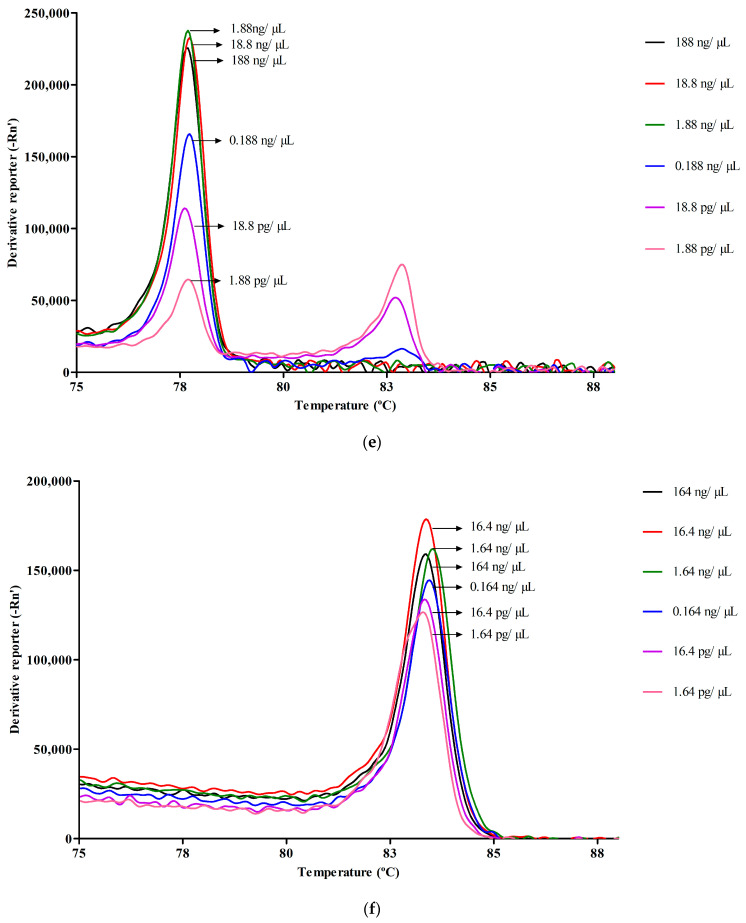

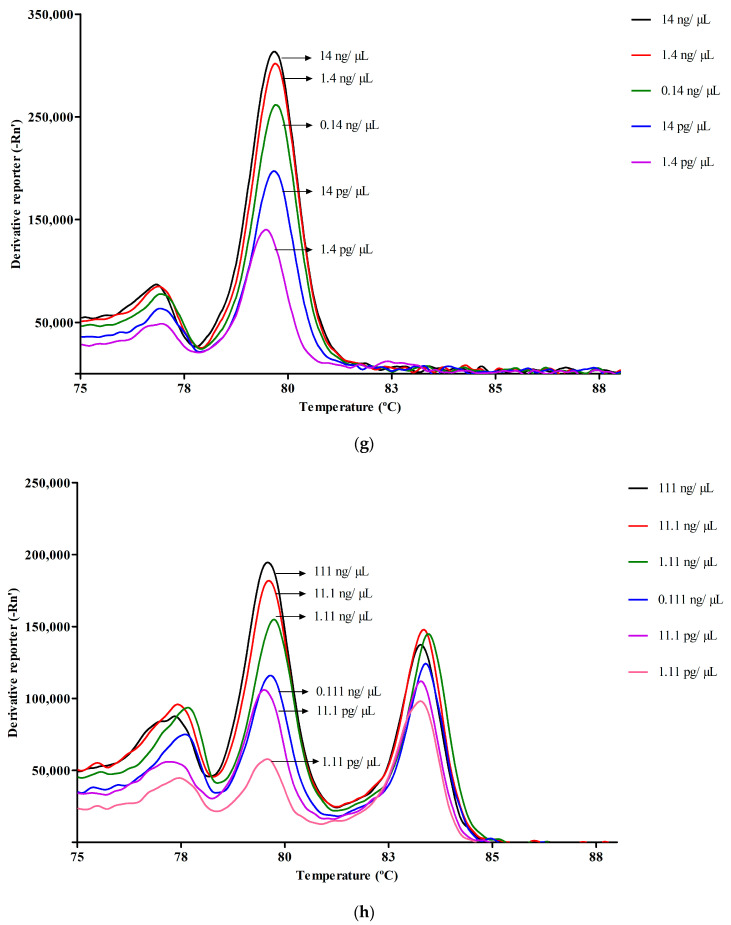
qPCR efficiency with dynamic range and coefficient of determination for *E. coli* O157 (**a**), *Salmonella* spp. (**b**) and *L. monocytogenes* (**c**) in simplex and multiplex (**d**). Curves for each situation were obtained by three replicates of ten-fold serial dilutions of a DNA extract from each pathogen, and a mixture of the three extracts and multiplex experiments. Graphic (**e**–**h**) represent the melting analysis of the same samples, respectively.

The amplification efficiency of the qPCR was also evaluated, in simplex and multiplex. The lower DNA concentration detected for *L. monocytogenes*, *Salmonella* spp. and *E. coli* O157 was 1.4, 1.6, 1.9 pg/µL respectively, see Figure 3a–c. Regarding the multiplex assay, a higher DNA concentration is needed, being the minimum concentration needed of 11 pg/µL (Figure 3d). The amplification efficiency was calculated after plotting the standard curves, and were determined to be 98.2%, 93.2%, 92.6% for *L. monocytogenes*, *Salmonella* spp. and *E. coli* O157 respectively; and 91.4% for the multiplex format. These results are shown in Figure 3. The influence of the DNA concentration in the melting peak are represented in Figure 3e–h, for each situation reported above, showing the decrease of the peak intensity with the decrease of DNA loaded in the qPCR reaction.

### 3.4. Methodology Evaluation

#### 3.4.1. Comparison of Expected and Obtained Results

The methodology for the target pathogens were evaluate with 44 samples with different contamination levels. The results are summarized in Table 4 for the four targets. All negative samples for one or more pathogens were correctly identified by qPCR, showing only the melting peak of NC-IAC and a Cq higher than 34 in a total absence of targeted bacteria. Considering only the samples positive when above the LoD, all positive samples were detected with the correct pathogen identification and none PD were observed, allowing a relative specificity, accuracy, of 100% and a Cohen’s k of 1 (Table 5).

#### 3.4.2. LoD Determination

**Figure 4:**
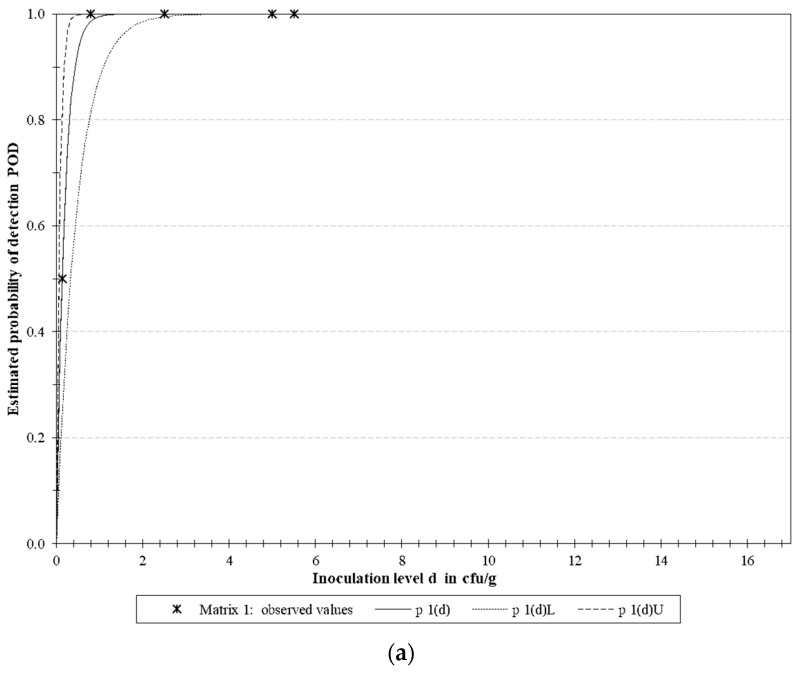

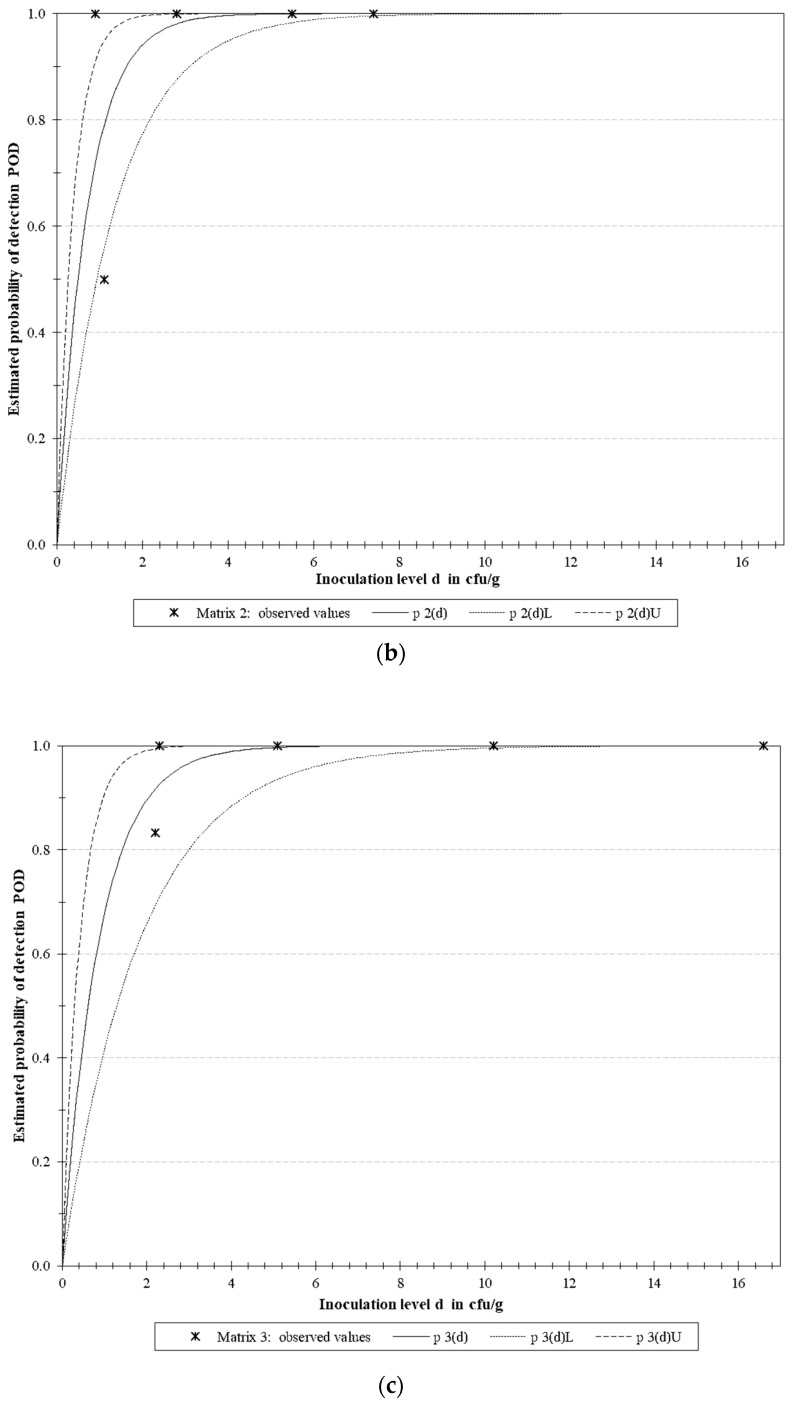

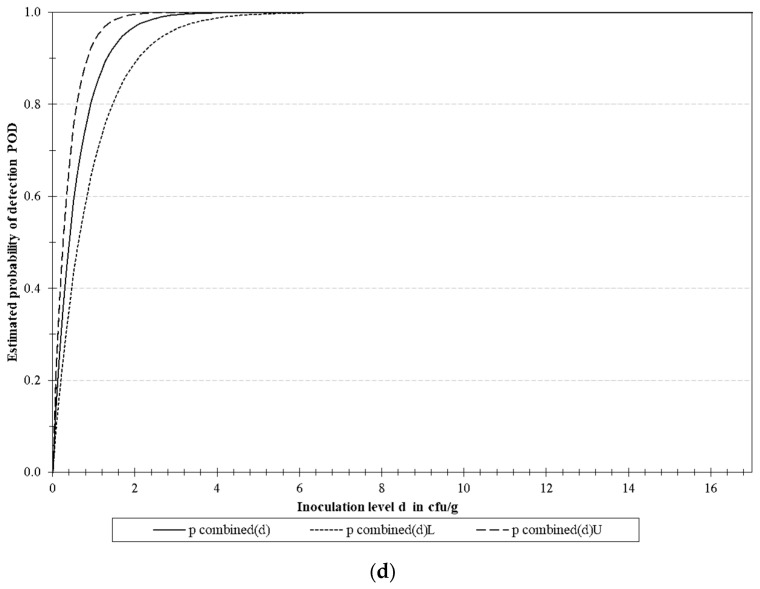
Graphical representation of the estimated probability of detection (POD) for *E. coli* O157 (**a**), *Salmonella* spp. (**b**), *L. monocytogenes* (**c**) and the combined detection of the three pathogens at the same time (**d**). Six samples inoculated with 5 different contamination levels were used for POD evaluation. The bacteria concentration used were 16.6, 10.2, 5.1, 2.3 and 2.2 CFU/25 g for *L. monocytogenes*, 7.4, 5.5, 2.8, 1.1 and 0.9 CFU/25 g for *Salmonella* spp. and 5.5, 5, 2.5, 0.8 and 0.2 CFU/25 g for *E. coli* O157. “*p* 1(d)” represents the POD, “*p* 1(d)L” the lower limit of the 95% confidence band, and “*p* 1(d)U” the upper limit of the 95% confidence band.

In total 30 samples were analyzed with five different levels of contamination to determine the LoD using POD analysis and results are represented in Table 6 and Figure 4. LoD 50% shown to be 0.1, 0.5 and 0.6 CFU/25 g and LoD 95% 0.6, 2.1 and 2.6 CFU/g for *E. coli* O157, *Salmonella* spp. and *L. monocytogenes*, respectively. The LoD of the multiplex detection in same sample was also evaluated, being the LoD 50% 0.4 and LoD 95% 1.7 CFU/25 g (Table 6).

## 4. Discussion

In the present study a multiplex qPCR methodology for the detection of *L. monocytogenes*, *Salmonella* spp. and *E. coli* O 157 taking advantage of the intercalating dye SYBR Green and melting analysis was developed. The first task accomplished, was the optimization of the enrichment medium, in order to improve the growth of all three target microorganisms, particularly *L. monocytogenes*, as it presents a lower growth rate compared with *Salmonella* spp. and *E. coli* O157, and for this reason the effort was to improve its growth, and prevent the excessive concentration of the two others bacteria. For this reason, the medium chosen was mTA10 with MOPS buffer. Despite no differences were observed between the use of KH_2_PO_4_/Na_2_HPO_4_ or MOPS to buffer mTA10 medium, previous studies have reported the capacity of MOPS to better recover stressed *L. monocytogenes* cells. Phosphate salts can have toxic effect to the bacterium when used in high concentration, however MOPS can be used in higher concentration, having an increased buffering capacity thus avoiding pH changes during enrichment, which can lead to growth delay [24,25]. The addition of glucose was evaluated in the new broth, even if this formulation promoted the growth *L. monocytogenes*, it was discarded, as it will also increase the numbers of *Salmonella* spp. and *E. coli* O157, thus generating a greater interference with the growth of *L. monocytogenes*. This observation is in agreement with previous studies [26,27]. Additionally, none of the supplements, or selective compounds, added to the medium were able to improve the growth of *L. monocytogenes*, having the opposite effect. The use of the different supplements exhibited a detrimental effect on the growth of *L. monocytogenes*. This phenomenon was particularly pronounced after the addition of Laked Horse Blood, and its combination with the other supplements tested. None them provided any significant improvement in the growth of the other two pathogens, but the minor effect observed in *L. monocytogenes*, combined with the fact that it is a slower-growing bacterium compared to *Salmonella* spp. and *E. coli* O157 [28], made us decide to use mTA10-MOPS without any supplement for multiplex detection.

The primers designed allowed the differentiation between the four melting peaks corresponding to the three pathogens and the NC-IAC. Three sets of primers were design and optimized to allow the detection of the three targets with highest sensitivity not losing specificity. The primers targeting the *rfbE* gene used in this methodology were already evaluated in a previous study dealing with the detection of *E. coli* O157 in food samples [21], showing high efficiency and accurate inclusivity and exclusivity. In the case of *actA* and *fimA* primers, they were specially designed for this methodology, and designed in a way which would allow the differentiation between the melting peaks generated in the qPCR. The *actA* is a virulence-associated gene, coding for a protein involved in the actin filament assembly and shows a high discriminatory power between *L. monocytogenes* strains and subtyping [29]; additionally, it has already been used in different qPCR approaches [30,31]. It is worth to mention that over the evaluation of the methodology, it was observed that *L. monocytogenes* (WDCM 00021), generated two peaks, one more predominant with the expected melting temperature (Tm) (79.47 ± 0.11 °C), and a smaller and wider one (76.30 ± 0.5 °C). The amplification products of this strain were analyzed by gel electrophoresis, but only one single fragment was visible (data not shown). This second peak appears close to the one of *E. coli* O157 (77.45 ± 0.13 °C), but were still clearly differentiated. The *fimA* is identified as one of the major virulence genes of *Salmonella* spp., wherein several qPCR reaction have been developed because of the discriminative capacity [13,32,33]. The melting temperature peak of this target (83.20 ± 0.13 °C) appear closer to the peak of the NC-IAC (82.67 ± 0.16 °C), but no interference with its detection was observed during the analysis, as clear peak was present for the identification of *Salmonella* spp. The Tm predicted by uMELT software and the obtained Tm by samples analysis present a similar pattern for all target. The adequacy of these genes for the detection of these pathogens, in simplex or multiplex, was well demonstrated in previous studies, and the primers designed in this study confirm those results, as all the strain tested were correctly identified.

In the current study, the primer concentration for *actA* had to be increased, with respect to the other targets in order to enhance the peak. This need could be due to the lower concentration of *L. monocytogenes* in the enrichment, or due to the preferential binding of SYBR Green to specific DNA fragments [34], what would agree with the fact that *fimA* had a bigger peak with a lower primer concentration.

A similar dynamic range was observed when tested DNA from pure bacterial cultures, as showed Figure 2, between 1–2 pg/µL. Regarding the multiplex detection of the three target simultaneously, the qPCR reaction show a 10 times higher LoD, as the *E.coli* O157 peak is less predominant than the other two target, some interference in this peak is present in lower range of DNA concentration. However, it is still possible to detect and identify correctly *L. monocytogenes* and *Salmonella* spp. when present 10 times less DNA concentration. The efficiency calculated for each situation was between the acceptable limits (90–110%) previously reported [35].

The LoD_50_ and LoD_95_ were only possible to calculate when intentionally obtained fractional positive detection of the targeted pathogens, generating false negatives samples, being below the LoD. For this reason, the SE, AP, PPV, NPV and k were calculated considering negative deviation only the samples above the LoD that gave negative results, and below this value, the rest of samples where considered negative agreement. The methodology proved to be reliable and sensitive as 100% of the obtained results were in concordance with the expected results in all parameters evaluated, and have comparable results to other studies using qPCR probe for multiplex pathogens detection [22,36,37]. The detection of the target in different concentration level in a same sample was successfully accomplished. Simultaneous detection may have advantages in terms of cost savings and rapidity. Most multiplex qPCR developed for the identification of different foodborne pathogens use hydrolysis probes [38,39] or high resolution melting analysis [15], which need appropriate reagents and a more complex downstream analysis, increasing the price in the first case or the time of the analysis in the second. On other hand, studies for multiplex real-time PCR without these approach, using only intercalating dyes have reported only the amplification of one or two target when an internal control is used [40,41], or when more pathogens are reported no internal control is employed [42,43]. The implementation of an internal control have been highly recommended by different authors, and the International Organization for Standardization (ISO) established this component as requirement for routine qPCR testing in food samples [44]. The use of an NC-IAC offer an unambiguous quality control, identifying when inhibition of the qPCR reaction occur, and avoiding false negative results [45,46]. A comparison against recent, previously published, similar methodologies, is provided in Table 7. Considering all these studies, the major advantages provided in our study rely on the implementation of the IAC for higher result confidence, along with the use of common fluorescent dye (SYBR Green) what avoids cost increase due to use of expensive fluorophores which, in addition, may need specific software.

With the novel methodology developed in this study, we were able to achieve the identification of three of the most problematic foodborne pathogens and the addition of the NC-IAC ensures the validity of the results, therefore allows its implementation in routine food analysis laboratories. The integration of the proposed method into routine testing could be performed in a straight forward manner. Two subsamples should be taken, one for microbiological quality assessment, and a second for pathogen detection as with this second matrix the three mentioned bacteria can be simultaneously detected. This would save time and resources (only one subsample is weighted, one single broth and one incubation temperature is needed, in comparison to three subsamples with different media and temperatures if analyzed independently), what overall would allow to reduce the cost of the analysis.

## 5. Conclusions

After the evaluation of the methodology, the application of qPCR with melting analysis proved to be a good alternative to probe-based qPCR, performing very robustly, producing reproducible, reliable data, and maintaining the performance in spiked sample, being a cost-effective methodology, as this approach make the analysis more economic compared to probe-based or formal high resolution melt analysis.

## Figures and Tables

**Table 1:** List of bacteria strain used to evaluate the specificity of the primers and qPCR reaction.

Bacterial Species	Source	N	*fimA*	*actA*	*rfbE*	IAC
*Salmonella* spp.	Mollusk (AMC 90, 238, 253)	3	+	-	-	-*
*Salmonella* spp.	Unknown (AMC 260, 261)	2	+	-	-	-*
*Salmonella* Oranienburg	Mollusk (AMC 28)	1	+	-	-	-*
*Salmonella* Veneziana	Mollusk (AMC 200)	1	+	-	-	-*
*Salmonella* Anatum	Proficiency test (AMC 60)	1	+	-	-	-*
*Salmonella* Enteritidis	Proficiency test (AMC 82)	1	+	-	-	-*
*Salmonella* Enteritidis	University of Bristol	1	+	-	-	-*
*Salmonella* Wentworth	Proficiency test (AMC 84)	1	+	-	-	-*
*Salmonella* Liverpool	Proficiency test (AMC 198)	1	+	-	-	-*
*Salmonella* Typhimurium	Proficiency test (AMC 96)	1	+	-	-	-*
*Salmonella* Typhimurium	Unknown (WDCM 00031)	1	+	-	-	-*
*Listeria monocytogenes*	Unknown (WDCM 00021)	1	-	+	-	-*
*Listeria monocytogenes*	Mollusk	8	-	+	-	-*
*Listeria monocytogenes*	Chestnut	6	-	+	-	-*
*Listeria monocytogenes*	Chicken	3	-	+	-	-*
*Listeria ivanovii*	WDCM 00018	1	-	-	-	+
*Listeria innocua*	WDCM 00017, CECT 5376, 4030, 1325, 1141, 2110	6	-	-	-	+
*Staphylococcus aureus*	WDCM 00034, 00033	2	-	-	-	+
*Staphylococcus* coagulase *+*	Proficiency test	1	-	-	-	+
*Campylobacter coli*	University of Minho	1	-	-	-	+
*Escherichia coli*	WDCM 00013, 00012	2	-	-	-	+
*Escherichia coli*	WDCM 00014	1	-	-	+	-

N: number of strains; IAC amplification allow to prove that any inhibition is present when the absence of amplification is observed in the rest of the targets. * The absence of IAC amplification is due to the amplification of at least one of the targeted bacteria. ”+/-” indicate positive or negative result by qPCR for the specified gene.

**Table 2:** Primer used in qPCR reaction.

Target Microorganism	Target Gene	Primers	Sequence 5′–3′	Concentration Used	Reference
*L. monocytogenes*	*actA*	actA F	TTAAGACTTGCTTTGCCAGAGAC	900 nM	[20]
		actA R	GGTGGTGGAAATTCGAATGAGC		
*Salmonella* spp.	*fimA*	fimA F	GCAATTGATGGCCTGTTAAACG	100 nM	In this study
		fimA R	AATTAACGCATTGCCAAAGATGC		
*E. coli* O157	*rfbE*	rfbE F	TCAACAGTCTTGTACAAGTCCAC	100 nM	[21]
		rfbE R	ACTGGCCTTGTTTCGATGAG		
NC-IAC *	-	NC-IAC F	TTAAGACTTGCTTTGCCAGAGAC	100 nM	[19]
		NC-IAC R	GGTGGTGGAAATTCGAATGAGC		

* NC-IAC correspond to the Non-Competitive Internal Amplification Control which allow the identification of amplification inhibition, to avoid false-negative results.

**Table 3:** Evaluation of the *L. monocytogenes* kinetic growth in different medium formulations.

	30 °C			35 °C		
	mTA10	mTA10-MOPS	mTA10-MOPS + Glucose	mTA10	mTA10-MOPS	mTA10-MOPS + Glucose
OD_600_ max	0.288 ± 0.004	0.261 ± 0.013	0.328 ± 0.010	0.242 ± 0.003	0.202 ± 0.006	0.292 ± 0.002
µmax	0.074 ± 0.004	0.078 ± 0.003	0.088 ± 0.001	0.089 ± 0.017	0.095 ± 0.003	0.085 ± 0.004
λ	14.166 ± 0.166	14.002 ± 0.157	14.770 ± 0.085	12.403 ± 0.143	13.090 ± 0.184	13.370 ± 0.079

OD_600_ max correspond to the maximum optical density, µmax represent the maximum specific growth rate, and λ the lag time in hours. Results were given by the model.

**Table 4:** Spiked samples.

Type of Sample	Contamination Level (CFU/25 g of Sample) *			N				
	*L. monocytogenes*	*S.* Typhimurium	*E. coli O157*		*actA*	*fimA*	*rfbE*	IAC
Infant Milk LoD	16.6	7	6	6	+	+	+	-
	10.2	6	5	6	+	+	+	-
	5	3	2	6	+	+	+	-
	2	1	1	6	+ (2ND)	+ (3ND)	+	-
	2	<1	<1	6	+	+	+(3ND)	-
Infant Milk	-	-	-	4	-	-	-	+
	9	6	4	2	+	+	+	-
	9.1	13	8	1	+	+	+	-
	9	6		1	+	+	-	-
		6	4	1	-	+	+	-
	9		4	1	+	-	+	-
	91	1.3 × 10^3^		1	+	+	-	-
	9		8.0 × 10^2^	1	+	-	+	-
		1.3 × 10^4^	8.0 × 10^3^	1	-	+	+	-
	9.1 × 10^3^	1.3 × 10^4^	8.0 × 10^3^	1	+	+	+	-

N: number of samples; * The contamination level correspond to concentration of bacteria inoculate before enrichment and was obtained by results of the plating in TSA for *S*. Typhimurium and *E. coli* O157 and TSYEA for *L. monocytogenes*. All Negative Deviations (ND) observed were below the LoD.

**Table 5:** Quality parameters from samples analysis.

Microorganism	Target	N	PA	PD	NA	ND	SE	SP	AC	PPV	NPV	k
*E. coli* O157	*rfbE*	44	32	0	12	0	100	100	100	100	100	1.00
*S*. Typhimurium	*fimA*	44	26	0	18	0	100	100	100	100	100	1.00
*L. monocytogenes*	*actA*	44	26	0	18	0	100	100	100	100	100	1.00

N: Number of samples, PA: positive agreement, NA: negative agreement, PD: positive deviation, ND: negative deviation, SE: relative sensitivity, SP: relative specificity, AC: relative accuracy, PPV: positive predictive value, NPV: negative predictive value, k: Cohen’s kappa. The positive and negative deviation were only considered when happening in samples above the LoD.

**Table 6:** Determination of the LoD based on fractional POD values.

Microorganism	Target	LoD_50_ *			LoD_95_ *		
		LoD	Lower Conf. Limit	Upper Conf. Limit	LoD	Lower Conf. Limit	Upper Conf. Limit
*E. coli* O157	*rfbE*	0.129	0.051	0.327	0.558	0.220	1.414
*S*. Typhimurium	*fimA*	0.488	0.257	0.930	2.111	1.109	4.018
*L. monocytogenes*	*actA*	0.613	0.292	1.288	2.649	1.261	5.567
Multiplex		0.398	0.253	0.627	1.721	1.093	2.710

* Limit of detection expressed as CFU/25 g.

**Table 7:** Comparison of previously published methods.

Reference	Pathogens Detected	Target	Fluorescent Dye	IAC	LoD and Matrix	Enrichment Time
Skerniškytė 2016 et al. [47]	Salmonella spp., Y. ente- rocolitica, L. monocytogenes and Campyl obacter spp. *Salmonella* spp. *Yersinia enterocolitica* *Listeria monocytogenes* *Campylobacter* spp.	*invA* *ystA* *hly* *16S_rrna*	SYTO 9	Yes	3.01 log CFU/mL 2.67 log CFU/mL 4.49 log CFU/mL 2.51 log CFU/mL Chicken rinses	-
Singh 2012 et al. [48]	*Listeria monocytogenes* *Salmonella* spp.	*hly* *invA*	SYBR Green	No	3 log CFU per mL 1 log CFU per mL Dried milk	6 h
Bundidamorn 2018 et al. [42]	Shiga toxin-producing *Escherichia coli* *Salmonella* spp. *Listeria monocytogenes*	*stx1,2* *invA* *hly*	EvaGreen	No	1 CFU/25 g raw chicken meat, raw pork, raw beef and fresh mung bean sprout	18 h
Kaynak 2016 et al. [49]	*Listeria monocytogenes* *Escherichia coli* O157:H7	*hly* *uid*	EvaGreen	No	<10 cells/mL Bovine and poultry meat	20 h
Xiao 2014 et al. [50]	*Salmonella* spp. *Listeria monocytogenes* *Staphylococcus aureus*	*hly,* *invA,* *Sa442*	EvaGreen	No	5 CFU/25 g Milk, chicken, beef, pork, egg	24 h
This study	*Listeria monocytogenes* *Escherichia coli* O157 *Salmonella* spp.	*actA* *rfbE* *fimA*	SYBR Green	Yes	2 CFU/25 g <1 CFU/25 g 2 CFU/25 g Infant formula	24 h

IAC: Internal Amplification Control. LoD: Limit of Detection.
