# Correction: Azinheiro et al. Multiplex Detection of *Salmonella* spp., *E. coli* O157 and *L. monocytogenes* by qPCR Melt Curve Analysis in Spiked Infant Formula. *Microorganisms* 2020, *8*, 1359

**DOI:** 10.3390/microorganisms9071482

**Published:** 2021-07-12

**Authors:** Sarah Azinheiro, Joana Carvalho, Marta Prado, Alejandro Garrido-Maestu

**Affiliations:** 1 Food Quality and Safety Research Group, International Iberian Nanotechnology Laboratory, Av. Mestre José Veiga s/n, 4715-330 Braga, Portugal; sarah.azinheiro@inl.int (S.A.); joana.carvalho@inl.int (J.C.); marta.prado@inl.int (M.P.); 2 College of Pharmacy/School of Veterinary Sciences, University of Santiago de Compostela, Campus Vida, E-15782 Santiago de Compostela, Spain

The authors would like to make the following correction to the published paper [1]:

There is a mistake in the primer sequences provided in Table 2 for the detection of *Salmonella* spp. The primers reported in the original version of the manuscript targeted the *fimA* as well, but they were not the ones used for the detection of *Salmonella* spp. The correct oligonucleotides designed in this study, targeting the *fimA* gene, are fimA F: CAC TAA ATC CGC CGA TCA AAC G and fimA R: TTC AGG ACG ATG GAG AAA GGC.
